# Effects of meal composition and meal timing on the expression of genes involved in hepatic drug metabolism in rats

**DOI:** 10.1371/journal.pone.0185520

**Published:** 2017-10-02

**Authors:** E. M. de Vries, J. E. Oosterman, H. M. Eggink, P. de Goede, S. Sen, E. Foppen, O. Boudzovitch-Surovtseva, A. Boelen, J. A. Romijn, S. E. laFleur, A. Kalsbeek

**Affiliations:** 1 Department of Medicine, Academic Medical Center, Amsterdam, the Netherlands; 2 Department of Endocrinology and Metabolism, Academic Medical Center, Amsterdam, the Netherlands; 3 Hypothalamic Integration Mechanisms, Netherlands Institute for Neuroscience, Amsterdam, The Netherlands; 4 Metabolism and Reward group, Netherlands Institute for Neuroscience, Amsterdam, The Netherlands; University of Lübeck, GERMANY

## Abstract

**Introduction:**

With chronotherapy, drug administration is synchronized with daily rhythms in drug clearance and pharmacokinetics. Daily rhythms in gene expression are centrally mastered by the suprachiasmatic nucleus of the hypothalamus as well as by tissue clocks containing similar molecular mechanisms in peripheral organs. The central timing system is sensitive to changes in the external environment such as those of the light-dark cycle, meal timing and meal composition. We investigated how changes in diet composition and meal timing would affect the daily hepatic expression rhythms of the nuclear receptors PXR and CAR and of enzymes involved in P450 mediated drug metabolism, as such changes could have consequences for the practice of chronotherapy.

**Materials and methods:**

Rats were subjected to either a regular chow or a free choice high-fat-high-sugar (fcHFHS) diet. These diets were provided *ad libitum*, or restricted to either the light phase or the dark phase. In a second experiment, rats had access to chow either *ad libitum* or in 6 meals equally distributed over 24 hours.

**Results:**

*Pxr*, *Alas1* and *Por* displayed significant day-night rhythms under *ad libitum* chow fed conditions, which for *Pxr* was disrupted under fcHFHS diet conditions. Although no daily rhythms were detected in expression of *CAR*, *Cyp2b2* and *Cyp3a2*, the fcHFHS diet did affect basal expression of these genes. In chow fed rats, dark phase feeding induced a diurnal rhythm in *Cyp2b2* expression while light phase feeding induced a diurnal rhythm in *Car* expression and completely shifted the peak expression of *Pxr*, *Car*, *Cyp2b2*, *Alas1* and *Por*. The 6-meals-a-day feeding only abolished the *Pxr* rhythm but not the rhythms of the other genes.

**Conclusion:**

We conclude that although nuclear receptors and enzymes involved in the regulation of hepatic drug metabolism are sensitive to meal composition, changes in meal timing are mainly effectuated via changes in the molecular clock.

## Introduction

The field of chronotherapy, the interdisciplinary science that aims to synchronize drug administration and biological rhythms, is receiving increasing attention. Drug clearance [1], toxicity [2] and pharmacokinetics all display clear changes over the light-dark cycle (reviewed in [3]). In addition, daily patterns are present in the manifestations and severity of many diseases, further highlighting the necessity for chronotherapy.

Daily rhythms in the mammalian body are mastered by the endogenous circadian (meaning approximately 24 h) clock in the suprachiasmatic nucleus of the hypothalamus (SCN) [4, 5] that is mainly entrained to the external environment by light. In addition to the central SCN clock, clocks in peripheral organs such as the liver are both entrained by the SCN via neural and endocrine pathways and by external stimuli such as nutrition [6]. Desynchronization between these central and peripheral clocks can alter the metabolic phenotype of an organism [7].

The first step of drug metabolism by the liver is carried out by phase I enzymes, the cytochrome p450 enzymes (P450). Of all the different isoforms of P450 enzymes present in the liver, the Cyp1-3 families are contributing to 70–80% of the metabolism of clinically prescribed drugs [8]. Transcription of P450 is regulated by the nuclear receptors constitutive androstane receptor (CAR) and the pregnane X receptor (PXR) [9]. In mouse liver, *Car* expression shows strong oscillating circadian patterns, whereas this is absent for *Pxr* [10]. In rodents, some but not all, P450 enzymes reveal daily variations in activity [11]. CYP3A4, the most important drug metabolizing P450 enzyme in humans, shows circadian oscillation *in vitro* [12]. In addition to the nuclear receptors, aminolevulinic acid synthase 1 (ALAS1), a rate-limiting enzyme in the production of heme, and cytochrome P450 oxireductase (POR) are key enzymes in P450 function. Both enzymes are regulated by the molecular clock [13–15].

The molecular clock consists of a sophisticated autoregulatory feedback loop with a period of approximately 24 hours. In short, the proteins CLOCK (*Circadian Locomotor Output Cycles Kaput)* and BMAL (brain muscle ARNT-like 1) form a dimer that regulates the expression of *Per* (period) and *Cry* (cryptochrome). As a heterodimer, PER and CRY inhibit the nuclear transcriptional activity of CLOCK and BMAL. By doing so they inhibit their own transcription which activates CLOCK:BMAL activity again [6, 16–18]. In addition to *Per* and *Cry*, the CLOCK:BMAL complex regulates a vast variety of metabolic genes such as *Pparα* (peroxisome proliferator-activated receptor alpha) and *Rev-erb* that link the clock to physiological processes. CLOCK:BMAL also regulates the expression of PAR bZIP proteins such as DBP (albumin D-site binding protein) which in turn is responsible for the circadian oscillations in *Car* expression [19, 20].

Contrary to the central SCN clock, the peripheral liver clock is particularly sensitive to changes in meal timing. When rats are subjected to restricted feeding in the resting (light) phase, and thus eat at an inappropriate time of day, clock gene expression in the liver is reversed and uncoupled from the master clock in the SCN [21–23]. In addition, also meal composition may affect the molecular clock system (reviewed in [24]). Since both meal timing and composition may affect the hepatic molecular clock, and the molecular clock regulates expression of genes involved in drug metabolism, it is evident that changes in eating behavior due to shiftwork, sleep disturbance and the 24/7 availability of highly palatable foods in our western society could have severe consequences for the practice of chronotherapy.

Therefore, the aim of our study was to address the following questions: 1) Do nuclear receptors and P450 isoform homologues to human P450 enzymes involved in drug metabolism display day-night oscillations?; 2) Does the ingestion of a free choice high-fat-high-sugar (fcHFHS) diet affect the hepatic expression of nuclear receptors and P450 enzymes? 3) Does meal timing affect these day-night oscillations? To this end, rats were subjected to either a chow or a fcHFHS diet [25]. Food was either available *ad libitum (ad lib)* or rats were only given access to food in the light period (light fed, or LF rats) or in the dark period (dark fed, or DF rats). To differentiate between the effect of the hepatic molecular clock and the timing of food intake, we conducted an additional experiment in which rats were either subjected to an *ad lib* chow diet or to a regimen where access to food was given in 6 predictable bouts per day (6-meals-a-day (6M) experiment). We determined daily patterns of hepatic gene expression by qPCR.

## Materials and methods

### Animals

Male Wistar rats (approximately 200 grams) were obtained from Charles River Laboratories International, Inc., Germany, and were group-housed upon arrival with three or four animals per cage under a 12:12 h light–dark (LD) cycle and with a room temperature of 20–21°C. All procedures were approved by the animal experimental committee of the Royal Netherlands Academy of Arts and Sciences (Permit number: NIN2014.21 and NIN2014.22).

### Diet experiment

**Fig 1:**
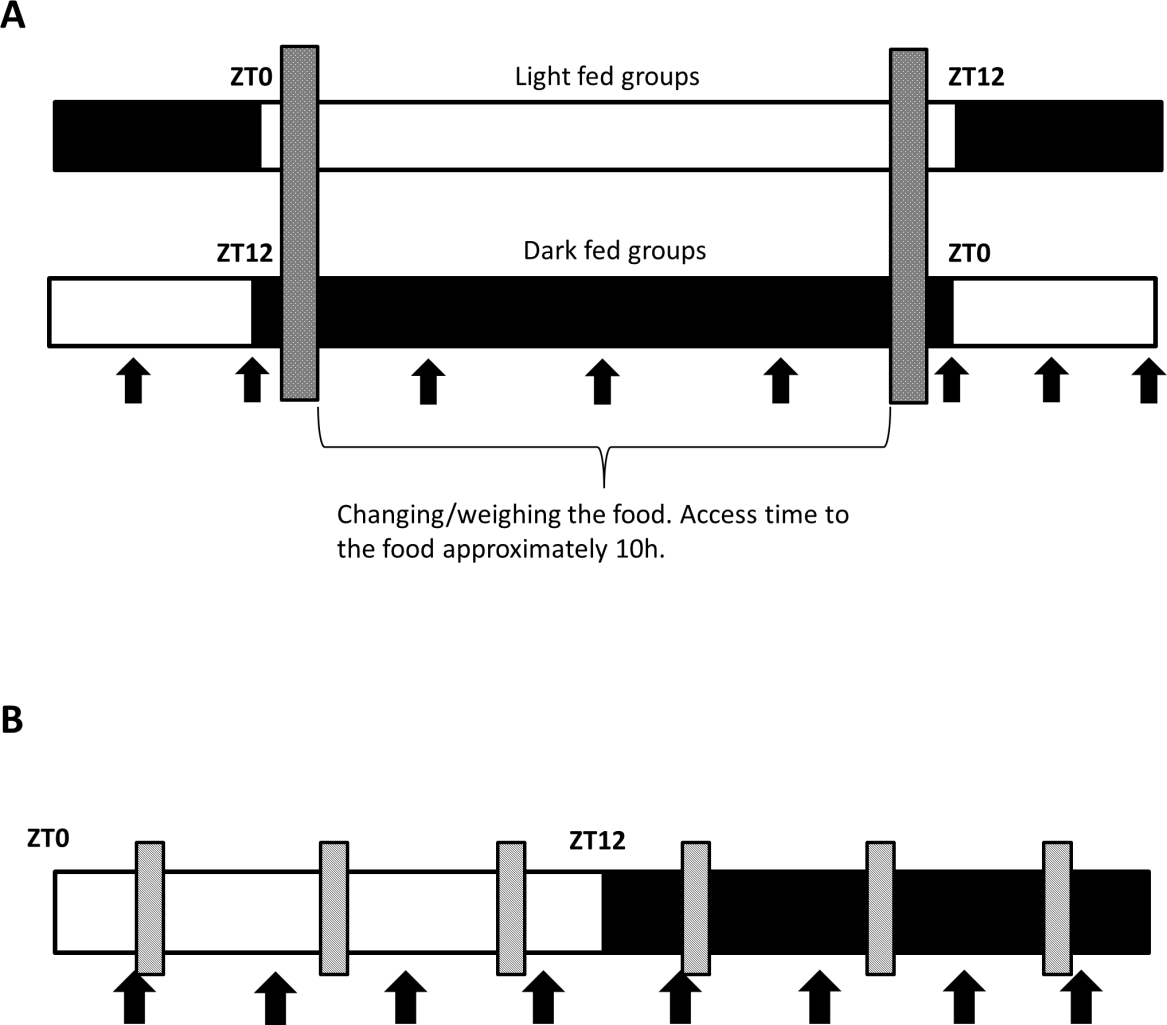
Schematic representation of the experiments. A) Diet experiment. Dark fed rats were on a reversed light/dark schedule. Grey bars indicate the weighing and changing of the food. In between rats had access to the respective diets (chow or fcHFHS). Arrows indicate the ZTs that the rats were sacrificed (ZT0, 3, 6, 9, 12, 15, 18, 21). B) Six meals experiment. Shaded bars represent food access for 10 minutes in 6 bouts per day. Arrows indicate the ZTs that the rats were sacrificed (ZT2, 5, 8, 11, 14, 17, 20, 23). White bars indicate the light phase while black bars represent the dark phase.

Rats that would receive food in the light period (light fed; LF) during the experiment were housed under regular LD conditions (lights on 7 AM (= Zeitgeber time (ZT) 0), lights off 7 PM), whereas rats that would receive food in the dark period (dark fed; DF), were housed under reversed LD conditions (lights on 7 PM, lights off 7 AM). Rats on regular and reversed LD schedules had an acclimatization period of 1 or 4 weeks, respectively. The *ad libitum (ad lib)* fed animals were randomized over the regular and reversed LD conditions. All rats had *ad lib* access to water and chow (Irradiated Global 18% protein rodent diet no. 2918, Harlan Nederland, Horst, the Netherlands) during the acclimatization period. The diet for the chow control groups consisted of chow and tap water. The free-choice high-fat-high-sugar (fcHFHS) diet consisted of a dish of saturated fat (beef tallow, ‘‘Ossenwit/Blanc de boeuf”, Vandermoortele, Belgium), and a bottle of 30% sucrose water (commercially available sugar in water), next to chow and tap water. Rats in the ‘*ad lib*’ groups had *ad libitum* access to the diet components for 24 h/day, whereas animals in the ‘LF’ and ‘DF’ groups only had access to food for 10 h starting 1 h into the dark or light period, respectively (Fig 1A). All groups had *ad lib* access to regular tap water. After 5 weeks on the diet, rats were sacrificed at 8 different time points (ZT 0, 3, 6, 9, 12, 15, 18, 21; n = 2–6 rats per time point for all the diet groups) by decapitation under carbon dioxide sedation. Liver tissue was dissected, snap frozen in liquid nitrogen and stored at -80°C until further use. For logistical reasons, the experiment was carried out in 5 different batches at separate times with consistent starting body weights and experimental regimen.

### Six meals experiment

Three days before the start of the feeding schedule, rats were individually housed and randomly assigned to the *ad lib* group or 6M group. Rats in the 6M group had access to food every 4 h for 12 min during the light phase and 11 min during the dark phase using an automated food dispenser (Fig 1B). The food dispenser consisted of a metal food hopper attached to the cage with vertical steel bars through which the rats could gnaw of their food. An automated sliding door prevented food access between the meals. Water was available *ad lib* for all rats. After 6 weeks on the diet, rats were sacrificed at 8 different time points (ZT2, 5, 8, 11, 14, 17, 20, 23; n = 4–8 rats per time point in the ad lib control group and n = 4–7 rats per time point in the 6-meals group) by decapitation under carbon dioxide sedation. Liver tissue was dissected, snap frozen in liquid nitrogen and stored at -80°C until further use.

### RNA isolation and qPCR

Total RNA from the rat liver was isolated using TriReagent (Ambion) and the Nucleospin RNA kit (Macherey-Nagel, Duren, Germany). Total RNA concentrations were determined using the DS-11 spectrophotometer (Denovix, Wilmington, Delaware USA). The cDNA synthesis was carried out using equal RNA input and the Transcriptor first strand cDNA synthesis kit (Roche molecular systems, Pleasanton, CA, USA). Quantitative PCR was performed using the Lightcycler 480 apparatus and the SensiFAST SYBR no-rox kit (Bioline, Londen, UK) and according to the MIQE guidelines [26]. Quantification was performed using the LinReg software [27]. Samples with a PCR efficiency that deviated more than 5% of the mean efficiency value of the assay were excluded. Calculated values were normalized by the geometric mean of 3 reference gene values (*Tbp*, *S18* and *Cyclophilin* [22]) which were selected to be the most stable among different groups. The raw data (not corrected for reference gene expression) can be found in S1 Data. The primers used are previously described [28] except for *Alas1*: Forward 5’-GCAACAGTCGAGTGCCAAAG-3’, Reverse 5’-ACGGTGTCGATCAGCAAACT-3’ and *Por*: Forward 5’-ATGGGGGACTCTCACGAAGA-3’, Reverse 5’-AAGCTGCTCTCTTTGACGGG-3’. The two nuclear receptors CAR and PXR were chosen since they are widely known for their role in the metabolism of exogenous compounds [9]. Cyp2b2 and Cyp3a2 were chosen because they are regarded as classic target genes of CAR and PXR. P450 isoforms were selected on homology to P450 isoforms that are important for the metabolism of some widely prescribed drugs [28], expression in the liver and literature [29].

### Data analysis and statistics

We used JTK cycle software to test whether genes were rhythmically expressed and to estimate the acrophase (ZT of the peak amplitude) [30]. Statistical analysis by Two-way ANOVA was performed with Graphpad prism version 7 (Graphpad, La Jolla, CA, USA) to test for overall group differences. All results are presented as means ± SEM except for JTK cycle acrophase estimation (mean). Results were considered statistical significant if p < 0.05. Line graphs show data in a double plot to better illustrate the rhythm.

## Results

### Daily rhythms in normal (ad lib chow) fed rats

**Fig 2:**
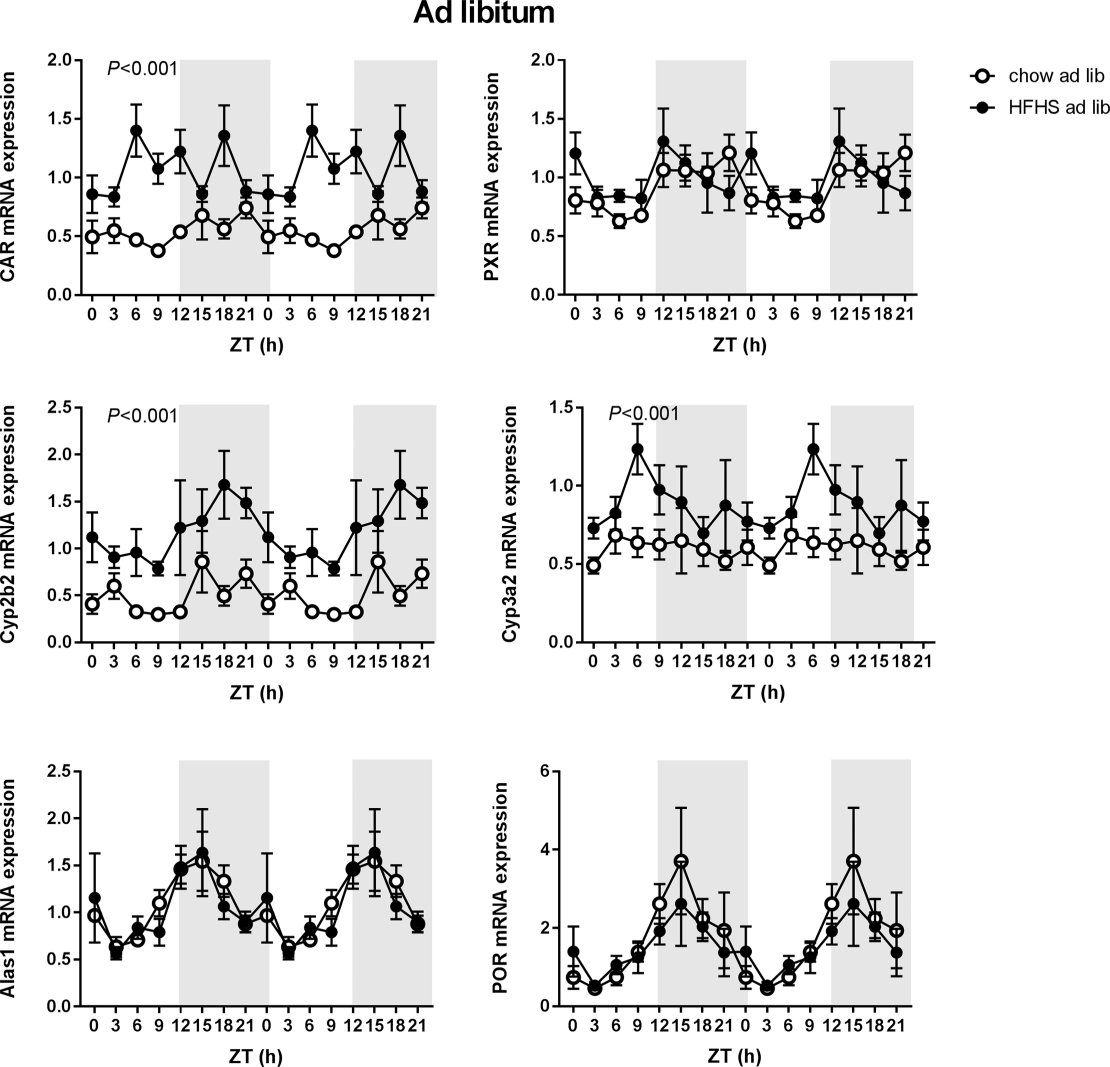
mRNA expression in *ad lib* fed chow and fcHFHS rats. Hepatic mRNA expression of the constitutive androstane receptor (*Car*), the pregnane X receptor (*Pxr*), CAR target gene *Cyp2b2*, PXR target gene *Cyp3a2*, 5'-Aminolevulinate Synthase 1(*Alas1*) and P450 oxireductase (*Por*). Open circles represent chow fed rats, closed circles represent fcHFHS fed rats (n = 2–6 rats per time point per diet group). The dark phase (lights off) is indicated in grey. P-values represent an effect of diet on mRNA expression as analysed by two-way ANOVA (using factors “diet” and “time”). Means ± SEM are presented in a double plot to better visualize the rhythms.

In *ad lib* chow fed rats, *Pxr* expression displayed significant day-night rhythmicity according to JTK cycle, whereas there was no significant daily rhythm for *Car* (Fig 2; Table 1). From the tested P450 enzymes, none displayed significant daily rhythmicity, whereas the P450 cofactors *Alas1* and *Por* did show a significant rhythmic expression.

**Table 1:** Data from the diet experiment analysed by JTK cycle.

**CHOW**	***ad libitum***			***dark fed***			***light fed***		
	P	acrophase	amplitude	P	acrophase	amplitude	P	acrophase	amplitude
CAR	—	—	0,090	—	—	0,110	<0.001	22.5	0,279
PXR	0.01	18	0,244	<0.001	16.5	0,358	<0.001	1.5	0,242
Cyp2b2	—	—	0,099	0.01	21	0,124	<0.001	9	0,243
Cyp3a2	—	—	0,042	—	—	0,031	—	—	0,061
Cyp1a2	—	—	0,142	—	—	0,148	—	—	0,088
Cyp2d2	—	—	0,091	—	—	0,101	—	—	0,062
Cyp2c6	—	—	0,079	—	—	0,091	0,000	0	0,144
Alas1	<0.001	15	0,362	<0.001	18	0,604	<0.001	4.5	0,262
POR	<0.001	15	0,980	<0.001	16.5	1,432	<0.001	4.5	0,945
**fcHFHS**	***ad libitum***			***dark fed***			***light fed***		
	P	acrophase	amplitude	P	acrophase	amplitude	P	acrophase	amplitude
CAR	—	—	0,169	<0.001	10.5	0,276	<0.001	0	0,383
PXR	—	—	0,127	<0.001	16.5	0,204	<0.001	22.5	0,301
Cyp2b2	—	—	0,318	0.002	18	0,300	—	—	0,222
Cyp3a2	—	—	0,145	0.045	13.5	0,241	—	—	0,188
Cyp1a2	—	—	0,103	—	—	0,176	—	—	0,109
Cyp2d2	—	—	0,031	—	—	0,149	—	—	0,115
Cyp2c6	0.018	0	0,031	—	—	0,082	—	—	0,046
Alas1	0,002	16	0,291	<0.001	15	0,324	—	—	0,121
POR	0,004	15	0,634	<0.001	16,5	1,419	<0.001	1,5	0,747

Acrophase (ZT at which expression peaks) in ZT is only given for genes that are rhythmically expressed (P<0.05).—not rhythmic according to JTK cycle.

### Effect of meal composition

fcHFHS feeding abolished the daily rhythm of *Pxr* mRNA expression, whereas it introduced a daily rhythm in *Cyp2c6* mRNA expression, as compared to a chow diet (Table 1). There were no effects of the fcHFHS diet on rhythmicity of *Alas1* and *Por* (Fig 2). Interestingly, the fcHFHS diet significantly increased the overall mRNA expression of *Car* (0.549 ± 0.219 for the chow fed group vs. 1.039 ± 0.354 for the fcHFHS fed group, expression in a.u.), its target gene *Cyp2b2* (0.499 ± 0.324 in the chow fed group vs. 1.137 ± 0.511 in the fcHFHS fed group), and the expression of the PXR target gene *Cyp3a2* (0.597 ± 0.209 for the chow fed group vs. 0.894 ± 0.322 for the fcHFHS fed group) (Fig 2).

### Effect of meal timing on the P450 pathway in chow fed rats

**Fig 3:**
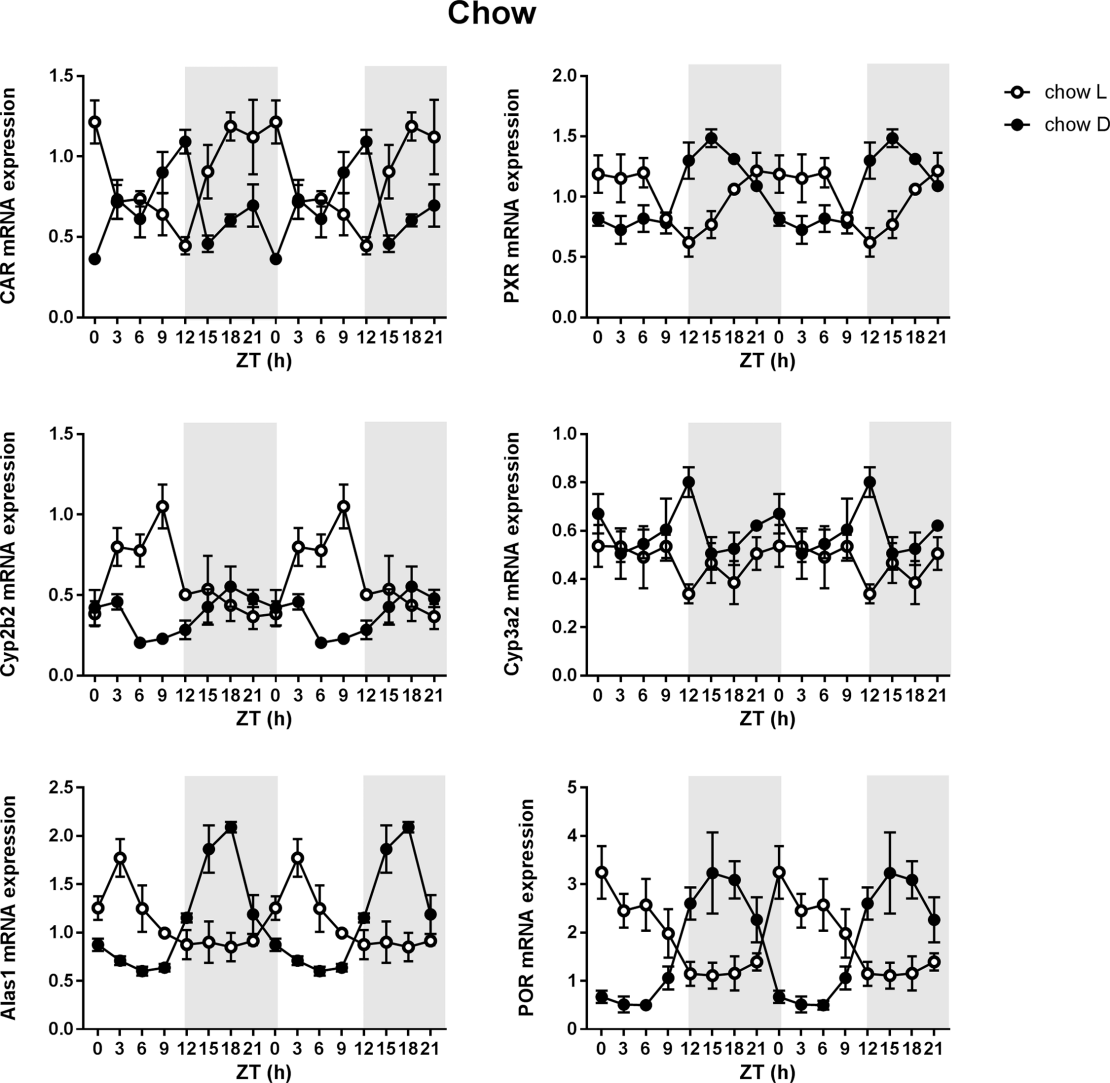
Effect of meal timing on mRNA expression in chow fed rats. Hepatic mRNA expression of the constitutive androstane receptor (*Car*), the pregnane X receptor (*Pxr*), CAR target gene *Cyp2b2*, PXR target gene *Cyp3a2*, 5'-Aminolevulinate Synthase 1(*Alas1*) and P450 oxireductase (*Por*). Open circles represent light fed rats, closed circles represent dark fed rats (n = 3–4 rats per time point per diet group). The dark phase (lights off) is indicated in grey. Means ± SEM are presented in a double plot to better visualize the rhythms.

In the chow fed rats, *Car* expression became rhythmic when rats were fed in the light phase (light fed; LF), with an acrophase of ZT 22.5 which was clearly shifted compared to the peak expression in dark fed (DF) rats (which was not rhythmic according to JTK cycle) (Fig 3). Also the phase of *Pxr* shifted in chow fed rats when rats were fed in the light phase (acrophase of ZT 1.5) compared to fed in the dark phase (acrophase of ZT 18) (Table 1, Fig 3). Rhythmicity in mRNA expression of the CAR target gene *Cyp2b2* was induced when feeding was restricted to the dark phase and the amplitude was even more pronounced when feeding was restricted to the light phase, with a clear shift of the acrophase between the LF (ZT 9) and the DF (ZT 21) rats (Table 1, Fig 3). Neither the expression of the PXR target gene *Cyp3a2*, nor the mRNA expression of other P450 enzymes *Cyp1a2*, *Cyp2d2* and *Cyp2c6*, were affected by meal timing (Table 1, graphic data only shown for *Cyp3a2*). On the other hand, the daily rhythms of *Alas1* and *Por* mRNA expression were shifted in LF rats compared to DF rats, (ZT 4.5 vs ZT 18 and ZT 4.5 vs ZT 16.5, respectively) (Table 1, Fig 3).

### Effect of meal timing on the P450 pathway in fcHFHS fed rats

**Fig 4:**
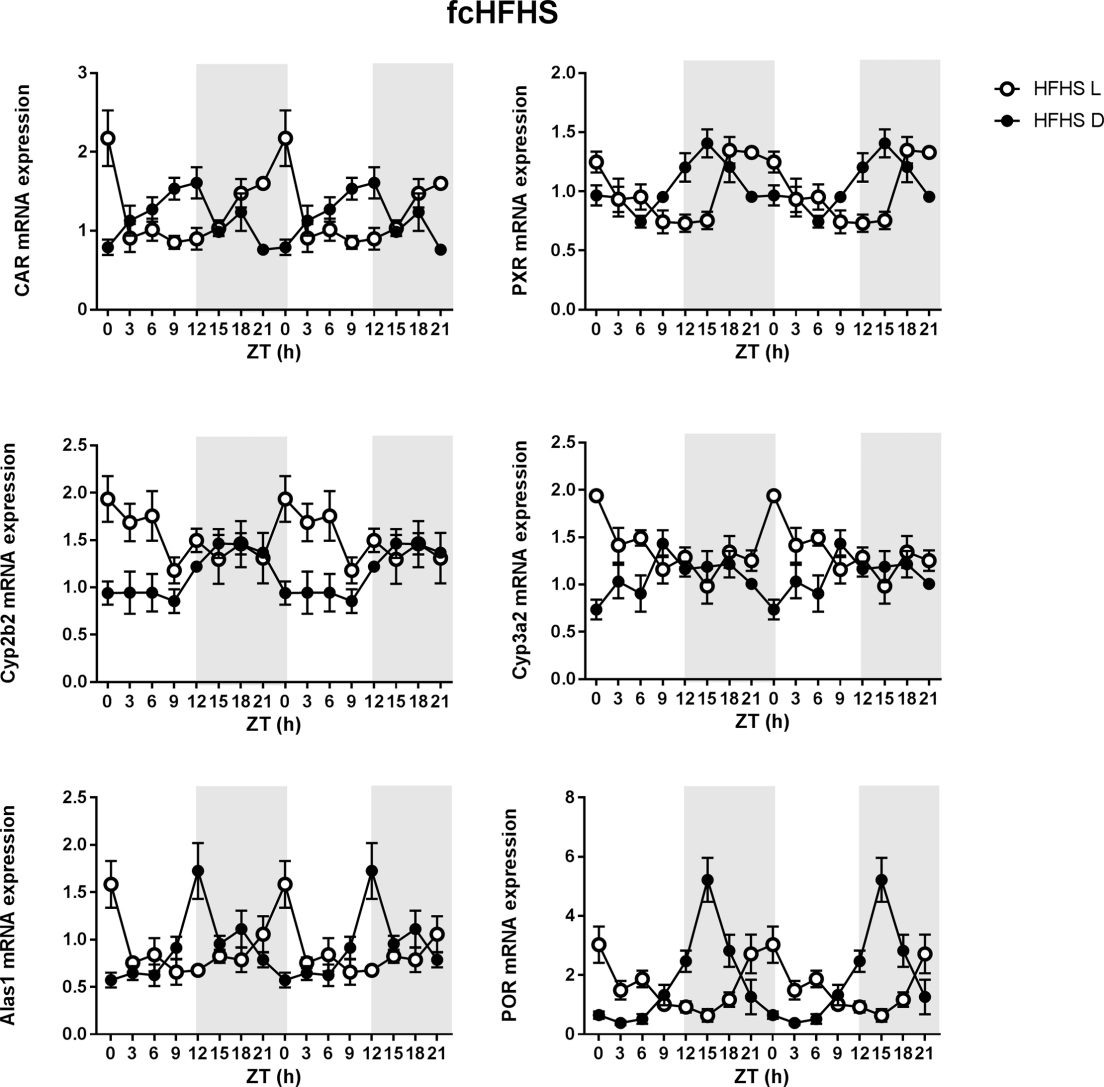
Effect of meal timing on mRNA expression in fcHFHS fed rats. Hepatic mRNA expression of the constitutive androstane receptor (*Car*), the pregnane X receptor (*Pxr*), CAR target gene *Cyp2b2*, PXR target gene *Cyp3a2*, 5'-Aminolevulinate Synthase 1(*Alas1*) and P450 oxireductase (*Por*). Open circles represent LF rats, closed circles represent DF rats (n = 3–4 rats per time point per diet group). The dark phase (lights off) is indicated in grey. Means ± SEM are presented in a double plot to better visualize the rhythms.

When rats were fed a fcHFHS diet, both light and dark phase feeding induced a significant day-night rhythm in *Car* expression with a clear shift in acrophase between the two groups (LF: ZT 0, DF: ZT 10.5) (Table 1, Fig 4). This shift was also visible in the expression of *Pxr* mRNA (LF: ZT 22.5, DF: ZT 16.5) (Table 1, Fig 4). A daily rhythm in *Cyp2b2* mRNA expression was present in DF rats. Light phase feeding clearly shifted the acrophase compared to dark phase feeding (Table 1, Fig 4). *Cyp3a2* expression in DF rats was rhythmic, while it was not in LF rats. No effects of meal timing were observed on the mRNA expression of other P450 enzymes *Cyp1a2*, *Cyp2d2* and *Cyp2c6* (Table 1). The *Alas1* rhythm was compromised in the LF rats, although a shift in peak acrophase was clearly visible between LF rats and DF rats. *Por* expression was shifted in LF (ZT 4.5) compared to DF rats (ZT 16.5) (Table 1, Fig 4).

### Effect of 6-meals-a-day feeding

**Fig 5:**
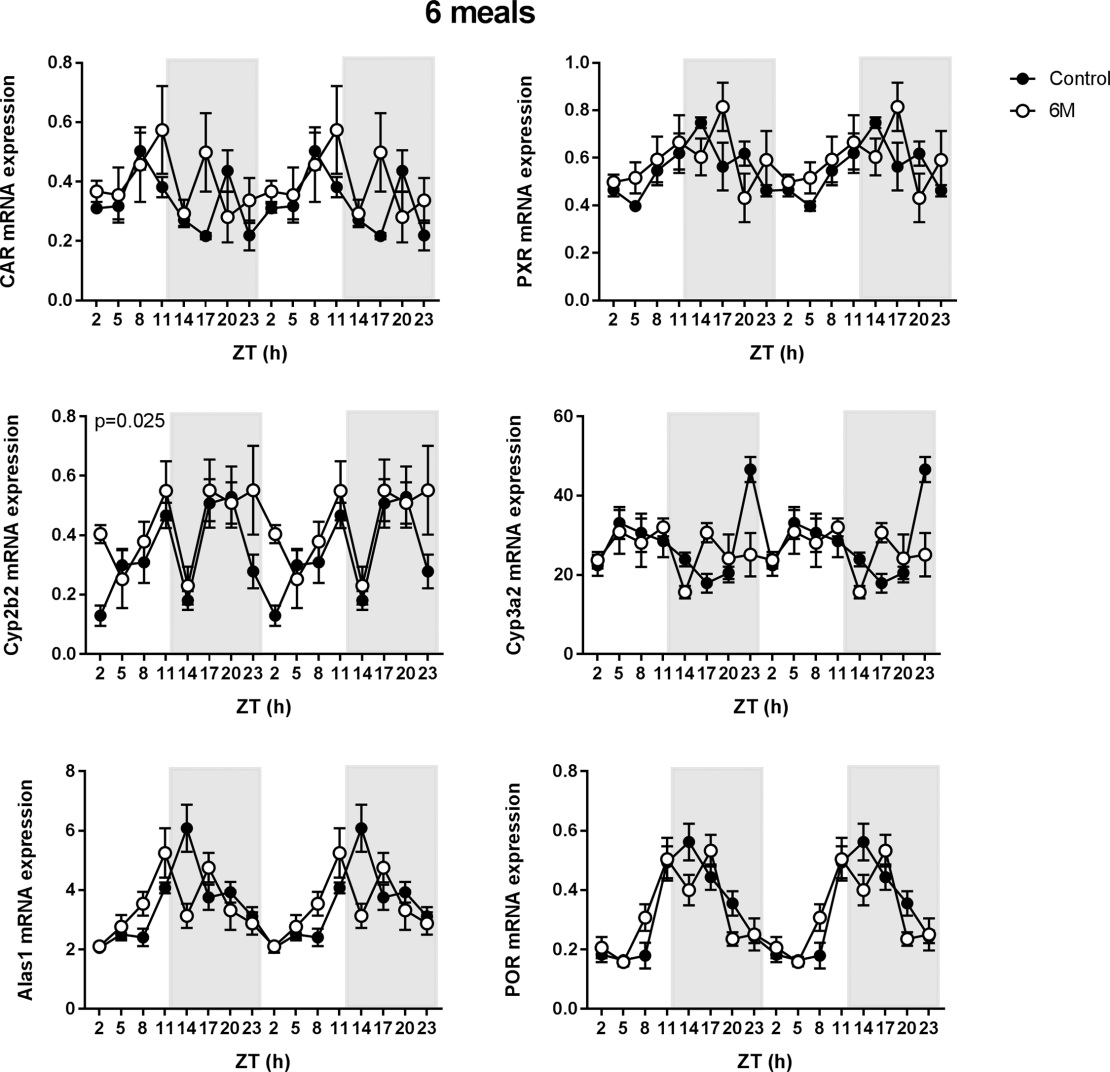
Effect of 6-meals-a-day feeding on P450 pathway mRNA expression. Hepatic mRNA expression of the constitutive androstane receptor (*Car*), the pregnane X receptor (*Pxr*), CAR target gene *Cyp2b2*, PXR target gene *Cyp3a2*, 5'-Aminolevulinate Synthase 1(*Alas1*) and P450 oxireductase (*Por*). Represented are chow fed rats that received *ad lib* feeding (closed black circles) or 6-meals-a-day (6M) feeding (open circles)(n = 4–8 rats per time point per diet group). The dark phase is indicated in grey (lights off). Means ± SEM are presented in a double plot to better visualize the rhythms. P-values represent the effect of the 6M feeding as measured with two-way ANOVA.

In concurrence with the results obtained from the diet experiment, *Car* expression did not display daily rhythmicity in *ad lib* chow fed rats, whereas *Pxr* was expressed rhythmically. The 6-meals-a-day feeding (6M) schedule did not affect *Car* expression, whereas it abolished the daily rhythm in *Pxr* expression (Table 2, Fig 5). Besides *Cyp2c6*, none of the tested P450 enzymes displayed a significant daily rhythm in the *ad lib* chow fed rats. The 6M schedule resulted in a loss of the significant *Cyp2c6* rhythm, whereas it had no effect on the other P450 enzymes (Table 2, Fig 5). *Alas1* and *Por* displayed significant daily rhythms in the *ad lib* fed rats, which was not affected by 6M feeding (Table 2, Fig 5).

**Table 2:** Data from the 6 meals experiment analysed by JTK cycle.

CHOW	*ad libitum*		* *	*6 meals*		
	P	acrophase	amplitude	P	acrophase	Amplitude
CAR	—		0,052	—		0,061
PXR	<0.001	15,0	0,136	—		0,069
Cyp2b2	—		0,109	—		0,039
Cyp3a2	—		2,874	—		2,972
Cyp1a2	—		0,042	—		0,043
Cyp2d2	—		2,350	—		2,290
Cyp2c6	0,02	6,0	8,301	—		4,172
ALAS1	<0.001	13,5	1,231	0,01	12,0	1,003
POR	<0.001	13,5	0,184	<0.001	13,5	0,145
Per1	<0.001	15,0	0,012	0,014	12,0	0,010

Acrophase (ZT at which expression peaks) in ZT is only given for genes that are rhythmically expressed (P<0.05).—not rhythmic according to JTK cycle.

## Discussion

Several aspects of hepatic drug metabolism display pronounced day-night variations, such as drug clearance and the associated drug toxicity [2]. In this paper, we describe the effects of meal timing and meal composition on the mRNA expression of nuclear receptors and P450 enzymes involved in hepatic drug metabolism in rats. We found that changes in meal timing but not meal composition induce profound phase shifts in daily rhythms of *Car*, *Pxr*, *Alas*1 and *Por* which are mediated by the hepatic molecular clock. These changes are likely to affect P450 mediated drug metabolism.

Phase I drug metabolism is carried out by cytochrome P450 enzymes. Circadian oscillations of a wide variety of P450 enzymes and nuclear receptors have been described in literature (reviewed in [11]). We show for the first time in rats that *Pxr* displays significant daily oscillations in *ad lib* chow fed conditions. None of the other nuclear receptors and P450 isoforms that we tested showed significant day-night rhythmicity. Surprisingly, although described as a highly oscillating gene, *Car* did not show a significant day-night rhythm when *ad lib* fed, although mRNA expression in the dark period tended to be higher than in the light period. The same was observed for *Cyp2b2* expression, a classic CAR target gene. None of the P450 enzymes, we tested, displayed a robust daily rhythm. Since the methodology to assess whether a gene displays a significant day-night oscillation greatly differs between studies (from simply testing a two time point day/night difference to the more advanced algorithms such as JTK cycle that we used), comparisons between studies are difficult. In addition, species differences might play a role. Using a cosine-wave pattern algorithm, Yang and coworkers found that nuclear receptors *Car*, *Shp* and *Rxr* were rhythmically expressed in mouse liver, whereas *Pxr* was not [10]. However, another study reported clear diurnal variation in *Pxr* expression in mouse liver [31]. Also in our study *Pxr* showed significant rhythmicity when rats were fed chow *ad lib*.

Heme production by 5-aminolevulinate synthase 1 (ALAS1) and electron transport by P450 oxidoreductase (POR), are essential for P450 activity. Several studies have shown a strong reciprocal interaction between components of the molecular clock and both heme production and POR activity [13–15], offering a posttranslational mechanism for the regulation of P450 enzyme activity. We measured *Alas1* and *Por* mRNA expression in *ad lib* chow fed rats and indeed found very strong day-night oscillations which suggests a functional day night difference in P450 mediated drug metabolism.

We investigated the effect of meal composition and meal timing on the cytochrome P450 enzymes. The effect of meal composition was investigated by feeding rats a fcHFHS diet, a diet intervention specifically designed to model the overconsumption of palatable food in our Western society, including the choice aspect [32, 33]. fcHFHS feeding did not blunt or induce the daily expression pattern of any of the genes tested. However, the fcHFHS diet did increase the overall expression of *Car*, *Cyp2b2* and *Cyp3a2*. Rats on the fcHFHS diet had a higher daily caloric intake than the chow groups, while in the second experiment the 6M groups had a lower caloric intake than the ad lib and 6M groups. In the second experiment basal expression of Car, Cyp2b2 and Cyp3a1 was not affected by the six-meal feeding regimen, we thus assume that the difference in the first experiment in basal expression of *Car*, *Cyp2b2* and *Cyp3a2* is due to the ingestion of fat and/or sugar. Literature on this subject is contradictory; a high fat diet for 13 weeks increased *Pxr* and *CYP3a2* in rats [34], whereas a high fat diet for 11 weeks decreased liver *Car*, *Pxr* and *Cyp3a2* expression in mice [35]. Dietary lipids do not have a uniform effect on P450 expression; ingestion of lard or corn oil by rodents induced *Cyp2e1*, *Cyp1a2*, *Cyp2b*, *Cyp3a* and *Cyp4a*, whereas this did not affect *Cyp2a1* and *Cyp2c11* expression [36]. Even less is known about the effect of high sucrose intake on hepatic P450 expression. An early study showed that high sucrose feeding decreased total CYP content [37], whereas another study in rodents showed that a high-fat-high-sucrose diet increased *Cyp1a2*, *Cyp1a1*, *Cyp2b10* and *Cyp2c29* expression while decreasing *Cyp3a11* expression [38]. Moreover, the mechanism(s) by which dietary components affect P450 expression are unclear at the moment, as well as the functional consequences for P450 function. Also, *Car* expression is also upregulated during fasting which is mediated via PPARα [28, 39] further complicating the interpretation of this data. We did not observe an effect of the fcHFHS diet on *Alas1* and *Por* mRNA expression.

Meal timing had pronounced effects on the daily rhythms of the studied genes. The daily pattern in mRNA expression of nuclear receptors *Car* and *Pxr*, and P450 cofactors *Alas1* and *Por* was completely shifted between rats that only had access to food in the light period compared to the dark period. Interestingly, this effect was essentially the same when rats were fed a fcHFHS diet except for *Pxr* which displayed a more moderate shift on a fcHFHS diet compared to the chow diet. These results reinforce the notion that timing of food intake rather than diet composition determines daily rhythms in gene expression. However, since hepatic clock gene expression also shifts with the restricted food intake in our timing experiment (Opperhuizen et al., 2016), it is difficult to confirm whether the shifts in gene expression observed in the different feeding schedules are caused by shifts in clock gene expression or feeding activity. To separate the influence of clock genes from food intake, we designed a second experiment in which we measured gene expression in the livers of *ad lib* fed rats and rats that received 6 meals a day, equally distributed over the 24 h. This paradigm removes the strong day-night rhythm in feeding activity, without abolishing or shifting the day-night rhythms in hepatic clock gene expression [40]. Interestingly, feeding rats according to a 6-meals-a-day schedule in this study only affected the rhythmic expression of *Pxr* and *Cyp2c6*, while *Car*, P450 enzymes, *Alas1* and *Por* expression did not differ between the *ad lib* and 6M fed rats. Expression of clock genes *Cry1* and *Dbp* was not affected in the 6M experiment (Eggink et al 2017, to appear in Chronobiology International). We can thus conclude that while some aspects of hepatic drug metabolism, such as *Pxr* and *Cyp2c6* expression seem to be sensitive for changes in meal composition and meal timing, other components are under the exclusive control of the hepatic molecular clock.

Taken together, the data from these experiments show that when food intake is restricted to an inappropriate time of day (for rodents that is eating during the light phase), the rhythmic expression of genes involved in hepatic drug metabolism is shifted, but the rhythmicity itself is not abolished. Surprisingly, when the influence of rhythmic meal timing is removed, as shown in the 6M experiment, daily gene expression of most aspects of P450-mediated drug metabolism still follows the expression pattern of *ad lib* fed animals, indicating that a factor, different from meal timing, affects circadian gene expression of these genes. The functional consequences of these meal timing-induced phase shifts for hepatic drug metabolism need to be further investigated, although the profound effect on *Alas1* and *Por* expression indicates that P450 mediated drug metabolism will be affected. Johnson et al recently showed that food intake during the light phase leads to shifts in acetaminophen toxicity [13], highlighting the role of food intake in chronotoxicity of drugs. Although direct extrapolation to clinical practice is challenging, especially since the nocturnal and diurnal difference between rodents and humans, these data show that disturbed meal timing can profoundly affect the diurnal pattern in gene expression of nuclear receptors and enzymes that are heavily involved in xeno- and endobiotic metabolism and might thus also have consequences for the practice of chronotherapy.

## Supporting information

S1 Data Raw qPCR data of the diet and 6-meals experiments. (XLS)
